# AR-Signaling in Human Malignancies: Prostate Cancer and Beyond

**DOI:** 10.3390/cancers9010007

**Published:** 2017-01-11

**Authors:** Michael T. Schweizer, Evan Y. Yu

**Affiliations:** 1 Division of Oncology, Department of Medicine, University of Washington, Seattle, WA 98109, USA; evanyu@u.washington.edu; 2 Fred Hutchinson Cancer Research Center, Seattle, WA 98109, USA

**Keywords:** prostate cancer, breast cancer, bladder cancer, renal cell carcinoma, pancreatic cancer, ovarian cancer, hepatocellular cancer, ovarian cancer, endometrial cancer, androgen receptor

## Abstract

In the 1940s Charles Huggins reported remarkable palliative benefits following surgical castration in men with advanced prostate cancer, and since then the androgen receptor (AR) has remained the main therapeutic target in this disease. Over the past couple of decades, our understanding of AR-signaling biology has dramatically improved, and it has become apparent that the AR can modulate a number of other well-described oncogenic signaling pathways. Not surprisingly, mounting preclinical and epidemiologic data now supports a role for AR-signaling in promoting the growth and progression of several cancers other than prostate, and early phase clinical trials have documented preliminary signs of efficacy when AR-signaling inhibitors are used in several of these malignancies. In this article, we provide an overview of the evidence supporting the use of AR-directed therapies in prostate as well as other cancers, with an emphasis on the rationale for targeting AR-signaling across tumor types.

## 1. Androgen Receptor Biology

Androgens, or male sex hormones, have a wide range of functions, including promoting the development of male secondary sexual characteristics, stimulating erythropoiesis, increasing metabolic rate, increasing bone density and stimulating libido [1]. In men, androgens are produced predominately by the testes, while the sole source of androgens in women are the adrenal glands. Consequently, women have considerably lower androgen levels compared to men. The normal physiologic function of androgens is a result of stimulating the androgen receptor (AR).

The AR is a member of the nuclear hormone receptor family of transcription factors, which also includes the estrogen receptor (ER), glucocorticoid receptor (GR), progesterone receptor (PR) and others [2,3]. Like the other nuclear hormone receptors, transcription of AR target genes is induced by the receptor binding androgenic ligands. Canonical AR-signaling involves a well-described series of events, including: (1) AR binding to androgens; (2) dissociating from heat-shock proteins; (3) translocating to the nucleus and the formation of AR homodimers; (4) binding to androgen response elements (AREs) within the promoter region of AR target genes; (5) recruitment of coactivators; and (6) transcription of target genes [4].

In addition to its normal physiologic role, prostatic adenocarcinomas remain dependent on AR-signaling even at later stages. Supporting the importance of AR to prostate cancer biology is the observation that AR target genes (e.g., *PSA*) are usually expressed even in men progressing on androgen deprivation therapy (ADT), with AR pathway alterations commonly observed in late stage disease [5]. This has served as the basis for ADT through medical and surgical castration, as well as the development of next generation AR-directed therapies like abiraterone and enzalutamide.

As our understanding of AR biology has improved, it has become apparent that the AR-signaling pathway can interact with a number of additional oncogenic signaling pathways, including those involved in promoting growth and resistance across a variety of tumor types (e.g., AKT/mTOR/PI3K, EGFR, HER2/Neu, Wnt) [5,6,7,8,9,10,11,12]. Interestingly, in spite of differences in consensus DNA binding motifs, AR is able to bind estrogen response elements and activate a transcriptional program similar to the ER—indicating that AR may be important mediator of breast cancer cell survival as well as other ER-dependent tumors [13,14]. The pleiotropic effects of AR-signaling raise the specter that targeting this pathway may have beneficial effects in a number of different cancers. In this review, we will outline the current evidence for testing AR-directed therapies in prostate, breast and other “non-hormonally” driven cancer like bladder, renal cell and pancreatic cancer, to name a few.

## 2. AR Targeting in Prostate Cancer

In 1941, Charles Huggins published his seminal paper describing the remarkable palliative effects of surgical castration in men with advanced prostate cancer [15]. We now understand that the beneficial effects of castrating therapy are a direct result of inhibiting AR-signaling, and as such targeting the AR has remained the backbone of prostate cancer therapy since the 1940s. As it stands, ADT is most often achieved through the use of luteinizing hormone releasing hormone (LHRH) agonists/antagonists as opposed to surgical castration; however, both achieve the same effect of lowering testosterone levels to the castrate range (i.e., <20–50 ng/dL) [16]. While ADT is initially highly effective, it does not represent a cure, and the vast majority of men with advanced prostate cancer will progress on ADT, developing castration-resistant prostate cancer (CRPC) [17,18].

Work over the last decade has shown that the AR remains a viable therapeutic target even in the castration-resistant setting. This was born out of the observation that AR target genes (e.g., *PSA*) are often expressed at high levels in patients with CRPC, and that expression of AR will go up in response to ADT [19,20]. It has also come to light that alternative sources of androgens, including those generated intratumorally, may also drive tumor growth in this setting [21,22]. As such, a number of next-generation AR-directed therapies have been developed to further inhibit AR-signaling, with abiraterone and enzalutamide both approved on the basis of Phase III data demonstrating improved overall survival compared to controls [23,24,25,26,27]. Abiraterone is a CYP17 inhibitor that targets extragonadal androgen biosynthesis in the tumor microenvironment and adrenal glands. Enzalutamide is an AR antagonist that is more effective than the first generation non-steroidal antiandrogens (e.g., bicalutamide, nilutamide). Because both of these agents target the ligand-AR interaction—abiraterone through ligand depletion and enzalutamide through antagonizing the AR-ligand binding domain—it is not surprising that numerous groups have documented evidence of cross-resistance between these drugs [28,29,30,31,32,33,34,35].

More recently, a number of studies have described mechanisms whereby AR-signaling is able to reemerge in spite of treatment with next generation AR-signaling inhibitors. Examples of these mechanisms include: AR amplification/overexpression, intratumoral androgen production, activation via feedback pathways (e.g., AKT/mTOR/Pi3K, HER2/Neu), activating AR ligand binding domain mutation, emergence of constitutively active AR splice variants and activation through other nuclear hormone transcription factors (e.g., GR) [6,7,19,21,36,37,38,39,40,41,42,43,44,45,46,47,48]. Several in depth reviews of these mechanisms have been published, and a detailed overview of their role in promoting resistance to AR-directed therapies is beyond the scope of this paper [3,20,49]. Suffice it to say, many ongoing drug development efforts are focused on developing more effective AR-directed therapies (e.g., drugs *not* targeting the ligand-AR interaction like EPI-506) or drugs to target key feedback pathways in selected populations (e.g., Akt inhibitors in patients with PTEN loss) [50,51,52].

## 3. Breast Cancer

### 3.1. AR in Breast Cancer

Like prostate cancer, breast cancer is a hormonally regulated malignancy. Indeed, shortly following the discovery that surgical castration was effective in men with advanced prostate cancer, Charles Huggins began exploring oophorectomy and adrenalectomy (with hormone replacement) as treatments for advanced breast cancer [53]. It is worth noting, however, that the German surgeon Albert Schinzinger was first credited with proposing oophorectomy as a treatment for breast cancer in the late 19th century [54]. While most hormonal-based therapies for breast cancer involve inhibiting estrogen receptor (ER)-signaling in hormone receptor positive subtypes, it has recently come to light that AR-signaling is likely an important modulator of breast cancer cell survival and may also be a viable target [55,56].

Several lines of clinical data support the biologic importance of AR-signaling in breast cancer, although AR positivity has been found to have variable prognostic impact across studies. Vera-Badillo, et al. conducted a systemic review of 19 studies that assessed AR immunohistochemistry (IHC) in 7693 patients with early stage breast cancer and found AR staining present in 60.5% of patients; interestingly, AR positivity was associated with improved overall survival (OS) [57]. The authors also found that AR positivity was more common in ER positive compared to ER negative tumors (74.8% vs. 31.8%, *p* < 0.001). However, it should be noted that AR antibodies used across studies was not consistent, nor was the cutoff defining “positivity”, making it difficult to draw firm conclusion regarding the overall prevalence of AR positivity across breast cancer subtypes.

Another study analyzing AR expression from tissue microarrays (TMAs) of 931 patients reported that 58.1% stained positive for AR, and that the association of AR with improved OS was only true for patients with ER positive tumors [58]. Apocrine tumors (ER negative, AR positive) with HER2 positivity associated with poorer survival, while AR did not appear to impact OS in triple negative breast cancer (TNBC) cases. A study by Choi and colleagues focused specifically on TNBCs (*n* = 559), found that AR was expressed in 17.7% of these cases, and that AR positivity was a negative prognostic feature. Two subsequent meta-analyses found that AR expression associated with better outcomes across tumor subtypes, however (i.e., ER positive, ER negative, and TNBC) [59,60].

### 3.2. Targeting AR in Breast Cancer

As mentioned, AR and ER are both nuclear hormone transcription factors and share a number of similar biologic features [55]. Upon binding their respective ligands, they undergo conformational changes, dissociate from heat shock proteins, dimerize and bind to DNA response elements where they promote transcription of target genes [3,61]. A number of studies have documented mechanisms whereby crosstalk between AR and ER exists, with most evidence supporting a model in which AR inhibits ER signaling through a variety of mechanisms—providing a biological basis for why AR positivity may associate with improved outcomes in ER positive breast cancers. AR is able to compete with ER for bindings at ER response elements (EREs), and transfection of MDA-MB-231 breast cancer cells with the AR DNA binding domain has been shown to inhibit ER activity [13]. Because the transcriptional machinery of both ER and AR involves a number of shared coactivator proteins, AR also likely inhibits ER activity through competing for binding of these cofactors [62,63]. Interestingly, there is also evidence that AR and ER can directly interact, with the AR N-terminal domain binding to the ERα ligand binding domain leading to decreased ERα transactivation [64].

The biologic action of AR in ER-negative breast cancers may differ significantly. AR is expressed in 12% to 36% of TNBCs, and in contrast to ER-positive breast cancers, data suggests that AR may be able to drive progression in some ER-negative cell lines [65,66,67,68,69,70,71]. Supporting the biologic importance of AR, and its viability as a therapeutic target, preclinical data has shown that AR antagonists (e.g., bicalutamide, enzalutamide) exert an anti-tumor effect in a number of ER-negative breast cancer models [65,67,72].

AR positive TNBCs are generally referred to as molecular apocrine tumors; however, more recent work has defined TNBCs on the basis of their molecular phenotype [73,74]. Work by Lehmann and colleagues have defined six subtypes of TNBC on the basis of their gene expression profiles: basal-like 1 and 2, immunomodulatory, mesenchymal, mesenchymal stem-like, and luminal androgen receptor (LAR) [74]. Interestingly, in spite of being ER-negative, the LAR subtype shares a gene expression signature similar to the luminal, ER-positive breast cancers. Chromatin immunoprecipitation (ChIP)-sequencing studies demonstrate that AR-binding events are similar to those of ERα in ER-positive breast cancer cell lines, indicating that AR may be able to substitute for ER in this context [14].

It should be noted that in addition to LAR tumors, other ER-negative, AR-positive breast cancer subtypes are sensitive to the effects of androgens [65,67]. Ni and colleagues have shown that in HER2-positive, ER-negative cell lines, AR mediates activation of Wnt and HER2 signaling in a ligand-dependent manner [67]. Further speaking to the importance of AR across breast cancer subtypes, Barton and colleagues reported that the next-generation AR antagonist enzalutamide is effective in several non-LAR TNBC subtypes. Interestingly, it has been shown that constitutively active AR splice variants (AR-Vs)—a well-described resistance mechanism in prostate cancer—are present in a large subset of breast cancer tumors, and that treatment of MDA-MB-453 cells (ER/PR-negative, HER2-negative, AR-positive) with enzalutamide can lead to the induction of AR-Vs [75]. The fact that a well-known resistance mechanism to AR-directed therapy appears relevant to breast cancer provides further support for the importance of AR-signaling in breast cancer.

### 3.3. Clinical Trials Targeting AR-Signaling in Breast Cancer

Early clinical data reported by Gucalp and colleagues supported AR as a therapeutic target in AR-positive, ER-negative/PR-negative breast cancers [76]. They conducted a single-arm, Phase II study testing bicalutamide 150 mg daily in patients with >10% nuclear AR staining. The primary endpoint was clinical benefit rate (CBR) defined as complete response (CR), partial response (PR) or stable disease >6 months. Overall, 51 of 424 (12%) screened patients were AR-positive as defined by the study. Twenty-eight patients were treated per protocol, with only 26 being evaluable for the primary endpoint. The study reported a clinical benefit in five patients (all with stable disease), which exceeded the predefined threshold (CBR = 4/28 patients) needed to justify further study.

A single-arm Phase II study testing enzalutamide in AR-positive TNBCs was more recently reported [77]. The primary endpoint was the CBR in “evaluable” patients which were defined as those with ≥10% AR staining and a response assessment. After testing 404 patient samples, 55% were found to have AR staining in ≥10% of cells. 118 patients were treated with enzalutamide, and 75 were “evaluable”. Of the evaluable patients, the CBR at 16 and 24 weeks was 35% and 29% respectively. The median progression free survival (PFS) in this group was 14 weeks. In patients with an AR gene signature (*n* = 56), clinical outcomes were numerically improved compared to the overall “evaluable” group and those lacking the gene signature (N = 62)—suggesting that further refinement of predictive biomarkers beyond AR IHC is necessary.

Abiraterone, an inhibitor of extragonadal androgen biosynthesis, has also been tested in breast cancer [78]. In a randomized Phase II trial, abiraterone was compared to the aromatase inhibitor exemestane or the combination. In contrast to the aforementioned studies, this study focused on ER-positive patients and did not require positive AR staining in order to enroll. The authors cited two reasons for not mandating AR-positivity: (1) upwards of 80% of ER-positive breast cancers are also positive for AR; and (2) inhibition of CYP17 will also decrease estrogen levels. The primary endpoint was PFS. A total of 297 patients were randomized between treatment arms, with 102 receiving exemestane, 106 receiving exemestane plus abiraterone and 89 receiving abiraterone. Of note, enrollment to the abiraterone monotherapy arm was discontinued early after a pre-specified analysis determined that futility conditions had been met. After a median follow up of 11.4 months, there was no difference in median PFS between when abiraterone was compared to exemestane (3.7 vs. 3.7 months, *p* = 0.437), or when abiraterone plus exemestane was compared to exemestane (4.5 vs. 3.7 months, *p* = 0.794). Of note, there was also no difference in PFS in the subset of patients with AR-positive disease.

Given that some studies have shown signs of activity for AR-signaling inhibitors, a number of additional trials are either planned or underway testing AR-directed therapies in breast cancer patients (Table 1). However, it seems likely that these agents will only be effective in a subset of patients, and as such, the development of predictive biomarkers will be critical. Whether the AR will prove to be a clinically important target in breast cancer remains to be seen, but evidence to date does support further testing of drugs designed to inhibit this oncogenic pathway.

## 4. Other Tumor Types

In addition to prostate and breast cancer, there are a number of other malignancies in which AR-signaling appears to play a role in driving tumor growth. As such, there are several ongoing clinical trials testing AR-directed therapies across an array of cancer types (Table 2). A brief overview of the rationale for targeting AR in these malignancies is provided below.

### 4.1. Bladder Cancer

In 2016, it is estimated that 58,950 American men will be diagnosed with bladder cancer compared to only 18,010 women [79]. Even after controlling for environmental risk factors (e.g., tobacco exposure) men still have a 3–4-fold increased risk of developing bladder cancer [80,81,82]. The observed epidemiologic differences in bladder cancer risk between the sexes points to the potential for sex steroid pathways to play a role in the pathogenesis of this disease [83]. Women have also been found to have a worse prognosis compared to men after adjusting for stage at presentation, further bolstering the case that underlying biologic differences between the sexes influencing outcomes [84].

Androgen receptor has been found to be variably expressed in urothelial carcinoma specimens, with AR staining present in 12% to 77% of patients [85,86,87,88,89]. In general, AR expression appears comparable in men and women [85,86]. There is no clear relationship between AR expression and clinical outcomes, and gene expression profiling studies do not demonstrate a clear relationship between AR expression levels and The Cancer Genome Atlas (TCGA) subtype [86,90,91].

Preclinical studies evaluating the effect of androgens and AR-signaling on urothelial carcinoma tumorigenesis have found that AR-signaling may promote tumor formation. In vitro siRNA studies have found that AR knockdown can lead to decreased tumor cell proliferation and increased apoptosis, possibly mediated through AR’s effect on *cyclin D1*, *Bcl-x(L)* and *MMP-9* gene expression [92]. In a separate set of experiments, mice engineered to not express AR in urothelial cells were found to have a lower incidence of bladder cancer following exposure to the carcinogen BBN [*N*-butyl-*N*-(4-hydroxybutyl)-nitrosamine] [93]. In vitro experiments found that this effect may be due to modulation of p53 and DNA damage repair. Studies have also implicated AR in modulating various other oncogenic signaling pathways (e.g., EGFR, ERBB2, β-catenin), offering more evidence for the importance of AR-signaling as it pertains to bladder cancer biology [94,95].

Kawahara and colleagues recently published a paper describing a series of in vitro and in vivo experiments in AR-positive and AR-null bladder cancer models [96]. They found that DHT increased AR-positive bladder cancer cell line viability and migration in culture, while AR antagonists (i.e., hydroxyflutamide, bicalutamide and enzalutamide) inhibited viability and migration. Similarly, apoptosis was decreased following exposure to DHT, and anti-androgens had the opposite effect. Importantly, enzalutamide was found to inhibit AR-positive bladder cancer xenograft growth in vivo. On the basis of these findings, two clinical trials have opened to test enzalutamide in patients with bladder cancer. One is testing enzalutamide monotherapy as a chemoprevention strategy in patients with non-muscle invasive bladder cancer [clinicaltrials.gov: NCT02605863], and the other is testing it in patients with advanced bladder cancer in combination with gemcitabine plus cisplatin [clinicaltrials.gov: NCT02300610].

### 4.2. Renal Cell Carcinoma

Androgen receptor is expressed in the distal and proximal tubules of normal kidneys and is expressed in approximately 15% to 42% of renal cell carcinomas (RCC) [97,98,99]. IHC studies correlating AR expression with clinical outcomes have not been consistent, with some reporting an association with decreased survival, while others have found that AR expression was correlated with a favorable pathologic stage and an overall favorable prognosis [97,100,101].

In a study evaluating AR transcript levels using real-time PCR, it was found that AR mRNA expression levels correlated with pathologic T stage and cancer specific survival. Multivariate regression analysis found AR transcript levels were independently associated with cancer specific survival. Of note, AR mRNA levels did not differ between sexes.

A more recent analysis of the TCGA data revealed that high AR protein and transcript levels was associated with improved overall survival in patients with clear cell RCC (the most common pathologic subtype), but not other histologic subtypes of RCC (i.e., papillary or chromophobe) [102]. Interestingly, in clear cell RCC cases they found that AR mRNA expression did not differ between men and women, but that AR protein expression was significantly higher in men. The authors concluded that AR might function as a tumor suppressor in this context.

In vitro experiments have reported that exposure to DHT causes proliferation in AR-positive RCC cells, while enzalutamide can reduce cell viability [103]. Other groups have found that AR may mediate tumor growth through activating HIF-2α/VEGF-signaling [104]. Preclinical studies have shown that enzalutamide can inhibit RCC cell migration and invasion by modulating HIF-2α/VEGF expression at the mRNA and protein levels. A neoadjuvant Pilot study testing enzalutamide in RCC patients is currently underway, with the primary goal to determine the effects of enzalutamide on RCC apoptosis and cellular proliferation [clinicaltrials.gov: NCT02885649].

### 4.3. Pancreatic Cancer

Although the incidence of AR expression is not well defined in pancreatic cancer, AR does appear to be expressed [105]. A number of in vitro/in vivo studies have tested the effects of antiandrogens and/or androgen deprivation in pancreatic cancer models, and have, for the most part, shown that inhibiting AR-signaling exerts anti-tumor effect [106,107,108,109,110,111,112,113]. Preclinical work has demonstrated that this effect may be mediated through IL-6, with a model whereby IL-6 activates AR-signaling via STAT3 and MAPK. Importantly, IL-6 has been shown to enhance pancreatic cell migration, an effect that is blocked through AR knockdown with an AR siRNA [114].

Greenway reported the results of a randomized trial comparing flutamide (a non-steroidal antiandrogen) vs. placebo (*n* = 49) in patients with both localized and metastatic pancreatic cancer [115]. It should be noted that histologic confirmation of pancreatic cancer was not required, and 32 included subjects were diagnosed on the basis of clinical presentation/imaging studies. This trial reported a median survival of 226 vs. 120 days in the flutamide and placebo groups, respectively (*p* = 0.079, Wilcoxon; *p* = 0.01, log-rank). Several other studies in patients with pancreatic cancer have not shown hormonal therapies to be beneficial, however [116,117,118,119,120,121].

Preliminary results from an ongoing Phase I study testing enzalutamide in combination with gemcitabine and nab-paclitaxel in patients with metastatic pancreatic cancer have recently been reported [122]. They have treated 19 patients, and report that 37% had tumor tissue positive for AR. Among 15 evaluable patients, two had a partial response and 13 had stable disease. Pharmacokinetic (PK) analyses did not find any evidence that enzalutamide altered the PK of either chemotherapeutic agent. Whether enzalutamide will prove to be an effective treatment for pancreatic cancer remains to be seen.

### 4.4. Hepatocellular Carcinoma

Androgen receptor appears to be expressed in subset of hepatocellular carcinomas (HCC), although, like pancreatic cancer, the incidence has not been well defined [123,124,125,126]. The majority of studies show that AR-positivity is associated with worse outcomes, including decreased progression free and overall survival as well as increased tumor size [126,127,128,129]. Studies have also linked AR-signaling with increased risk of developing hepatitis B and C related HCC [130,131,132,133]. AR has been found to promote HCC growth, migration and invasion in several preclinical studies, possibly through increasing oxidative stress and DNA damage, as well as suppressing p53 [134,135,136]. In vitro and in vivo studies targeting AR with either AR-siRNA or ASC-J9 (an AR protein degrader) resulted in decreased tumor growth [134]. A randomized Phase II study testing enzalutamide vs. placebo in HCC is currently underway [clinicaltrials.gov: NCT02528643].

### 4.5. Ovarian Cancer

In 1998, Risch hypothesized that epithelial ovarian cancers may develop as a result of androgens stimulating epithelial cell proliferation, and as it stands, a number of lines of evidence support the role for AR-signaling in the pathogenesis of the disease [137,138]. AR is highly expressed in ovarian cancers, with approximately 44% to 82% of tumors staining positive for AR [139,140,141]. Polycystic ovarian syndrome (PCOS), and its resultant hyperandrogenic state, are associated with hyperplastic and metaplastic changes in the surface epithelium of the ovaries, and women with ovarian cancer are more likely to have a history of PCOS compared to control cases [142,143]. The use of exogenous androgens (i.e., danazol, testosterone) has been associated with a >3-fold increased risk of developing ovarian cancer [144]. Preclinical models also support the hypothesis that androgens play a role in the development of epithelial ovarian cancers, with a number of oncogenic signaling pathways implicated in this process (e.g., TGF-β, IL-6/IL-8, EGFR) [138,145,146,147]. However, as it stand, the prognostic impact of AR expression in epithelial ovarian cancers is not clear [138].

A handful of clinical trials testing AR-signaling inhibitors in women with ovarian cancer have been completed, with no clear signs of activity. A single-arm Phase II study testing flutamide in ovarian cancer patients progressing on platinum chemotherapy has previously been reported [148]. Out of 68 women enrolled, only two objective responses (one complete and one partial response) were observed. In a second single-arm Phase II study, flutamide was given to 24 ovarian cancer patients who failed chemotherapy and only one partial response was observed [149]. Finally, in a single-arm Phase II study, Levine and colleagues treated 35 women with ovarian cancer who were in second or greater complete remission with bicalutamide and goserelin (LHRH agonist) [150]. This trial failed to meet the pre-specified metric to justify further studies testing this regimen, which was arbitrarily set at median PFS >13.5 months. More recent preclinical work has shown that enzalutamide is able to significantly inhibit the growth of ovarian cancer xenografts [151]. On this basis, a Phase II study has been launched to test enzalutamide in women with AR-positive, advanced ovarian cancer [clinicaltrials.gov: NCT01974765].

### 4.6. Endometrial Cancer

Similar to prostate and breast cancer, endometrial cancers are hormonally dependent, and hormonal agents targeting ER-/PR-signaling are options for select patients [152]. Given the similarities to breast and prostate cancer, Tangen and colleagues sought to explore the potential for targeting AR-signaling in advanced endometrial cancer [153]. They found that the majority of hyperplastic endometrial specimens evaluated (93%) had evidence of AR expression. This number decreased in primary tumors, and high-grade tumors (i.e., grade 3) were found to express less AR than low-grade tumors (i.e., grade 1) (53% vs. 74%). Metastatic specimens from 142 patients revealed AR expression in 48% of samples. On multivariate analyses, AR status did not provide additional prognostic value, however. Short-term cell culture experiments demonstrated that cell proliferation was inhibited by enzalutamide, and stimulated by the synthetic androgen R1881, providing justification for a Phase II study testing enzalutamide in combination with carboplatin and paclitaxel [clinicaltrials.gov: NCT02684227].

### 4.7. Mantle Cell Lymphoma

Mantle cell lymphoma shows a male predominance, and interestingly, male sex appears to associate with higher mortality based on a retrospective SEER analysis [154]. While it is not clear what underlies the poor outcomes in men with mantle cell lymphoma, AR is expressed across an array of hematopoietic cells, and may account for gender differences in the function of platelets and the immune system [155,156,157]. Furthermore, in contrast to other lymphomas, *AR* appears to be hypomethylated in mantle cell lymphoma—indicating that epigenetic silencing of *AR* gene expression may not be present in mantle cell lymphoma [158,159]. To our knowledge, large studies examining AR protein expression in mantle cell lymphoma samples have not been conducted. On the basis of these observations a pilot study was recently launched to assess the clinical effects of enzalutamide in patients with mantle cell lymphoma [clinicaltrials.gov: NCT02489123].

### 4.8. Salivary Gland Cancer

AR is expressed in the majority of lacrimal gland ductal carcinomas, and as a result AR staining is often used as part of the workup to confirm the diagnosis [160,161,162,163,164,165,166]. To date, there have been a handful of case reports/series documenting favorable outcomes in patients with salivary gland cancers treated with AR-directed therapies. A small case series (*n* = 10) reported a clinical benefit when ADT—most often single agent bicalutamide—was given to patients with salivary ductal carcinoma, with 50% of patients experiencing clinical benefit (i.e., stable disease, *n* = 3; partial response, *n* = 2) [167]. A case report has also reported favorable outcomes when ADT was combined with radiation therapy in a patient with AR-positive salivary gland cancer [168]. A single arm Phase II study testing enzalutamide in AR-positive salivary gland cancers is ongoing [clinicaltrials.gov: NCT02749903].

## 5. Conclusions

AR signaling is involved in a number of normal physiologic processes, and there is varying levels of evidence for its role in promoting cancer growth and progression across an array of malignancies. To date, prostate cancer remains the only malignancy with Level 1 evidence supporting the use of AR-directed therapies as an integral part of its treatment paradigm. However, mounting preclinical, epidemiologic and early phase clinical trial data support the further exploration of these drugs in diseases as varied as breast and salivary gland cancers, and it is likely that in the ensuing decade next generation AR-directed drugs will extend their reach beyond prostate cancer.

## Figures and Tables

**Table 1:** Ongoing studies testing AR-directed therapies in breast cancer. Abi, abiraterone; Enza, enzalutamide; AR, androgen receptor; AE, adverse event; MTD, maximum tolerated dose; CR, complete response; PR, partial response; and SD, stable disease.

Indication	Therapeutic Agent(s)	Disease State	Study Phase	Sample Size	Primary Endpoint	NCT Number
Breast cancer	Enza, enza + anastrozole, enza + exemestane, enza + fulvestrant	Advanced	Phase I	101	Safety	NCT01597193
Breast cancer	Enza + exemestane	Advanced	Phase II	247	Progression free survival	NCT02007512
Triple-negative breast cancer	Enza + paclitaxel vs. placebo + paclitaxel	Advanced	Phase III	780	Progression free survival	NCT02929576
AR positive, triple-negative breast cancer	Enza + taselisib	Advanced	Phase I/II	73	MTD	NCT02457910
AR positive, triple-negative breast cancer	Enza + paclitaxel	Localized (neoadjuvant)	Phase II	37	Pathologic complete response and minimal residual disease	NCT02689427
HER2 positive and AR positive breast cancer	Enza + trastuzumab	Advanced	Phase II	80	Clinical benefit rate: combined CR, PR and SD	NCT02091960
AR positive, triple-negative breast cancer	Enza	Localized (adjuvant)	Phase II	200	Treatment discontinuation rate	NCT02750358
AR positive, triple-negative breast cancer	Enza	Advanced	Phase II	118	Clinical benefit rate: combined CR, PR and SD	NCT01889238
Breast cancer	VT-464	Advanced	Phase I/II	110	MTD	NCT02580448
Breast cancer	Abi	Advanced	Phase I/II	74	MTD, causality of AEs, and clinical benefit rate: combined CR, PR and SD	NCT00755885
ER positive HER2 negative breast cancer	Abi	Advanced	Phase II	299	Progression free survival	NCT01381874
HER2 negative breast cancer	Abi	Advanced	Phase II	31	Clinical benefit rate: combined CR, PR and SD	NCT01842321
ER positive HER2 negative breast cancer	Abi vs. anastrozole	Localized (neoadjuvant)	Phase II	--	Gene expression differences	NCT01814865
AR positive breast cancer	Orteronel	Advanced	Phase II	86	Response rate: complete and partial responses	NCT01990209
Breast cancer	Orteronel	Advanced	Phase I	8	Safety, recommended Phase II dose, and decrease in estradiol levels	NCT01808040

**Table 2:** Ongoing studies testing AR-directed therapies in cancers other than breast or prostate cancer. Enza, enzalutamide; AR, androgen receptor; and MTD, maximum tolerated dose.

Indication	Therapeutic Agent(s)	Disease State	Study Phase	Sample Size	Primary Endpoint	NCT Number
Endometrial cancer	Enza + carboplatin + paclitaxel	Advanced	Phase II	69	Safety/objective tumor response	NCT02684227
Hepatocellular carcinoma	Enza vs. placebo	Advanced	Phase II	144	Overall survival	NCT02528643
Hepatocellular carcinoma	Enza vs. Enza + sorafenib	Advanced	Phase I/II	73	Safety	NCT02642913
Non-muscle invasive bladder cancer	Enza	Localized (chemoprevention)	Phase II	50	Recurrence rate	NCT02605863
Bladder cancer	Enza + cisplatin + gemcitabine	Advanced	Phase I	24	MTD	NCT02300610
AR positive ovarian cancer	Enza	Advanced	Phase II	58	Response rate: complete and partial responses	NCT01974765
Pancreatic cancer	Enza + gemcitabine + nab-paclitaxel	Advanced	Phase I	38	MTD	NCT02138383
Renal cell carcinoma	Enza	Localized (neoadjuvant)	Pilot/Phase 0	20	Cell proliferation and tumor apoptosis	NCT02885649
Mantle cell lymphoma	Enza	Advanced	Pilot/Phase 0	20	Response rate: complete and partial responses	NCT02489123
AR positive salivary cancer	Enza	Advanced	Phase II	45	Response rate: complete and partial responses	NCT02749903
